# Dicalcium Phosphate Dihydrate Mineral Loaded Freeze-Dried Scaffolds for Potential Synthetic Bone Applications

**DOI:** 10.3390/ma15186245

**Published:** 2022-09-08

**Authors:** Neelam Iqbal, Thomas Michael Braxton, Antonios Anastasiou, El Mostafa Raif, Charles Kai Yin Chung, Sandeep Kumar, Peter V. Giannoudis, Animesh Jha

**Affiliations:** 1 School of Chemical and Process Engineering, University of Leeds, Leeds LS2 9JT, UK; 2 Institute of Medical and Biological Engineering, University of Leeds, Leeds LS2 9JT, UK; 3 Department of Chemical Engineering and Analytical Science, University of Manchester, Manchester M1 3AL, UK; 4 Faculty of Medicine and Health, School of Dentistry, University of Leeds, Leeds LS2 9JT, UK; 5 Academic Department of Trauma and Orthopaedic Surgery, School of Medicine, University of Leeds, Leeds LS2 9JT, UK

**Keywords:** dicalcium phosphate dihydrate, chitosan, osteogenesis

## Abstract

Dicalcium Phosphate Dihydrate (DCPD) mineral scaffolds alone do not possess the mechanical flexibility, ease of physicochemical properties’ tuneability or suitable porosity required for regenerative bone scaffolds. Herein, we fabricated highly porous freeze-dried chitosan scaffolds embedded with different concentrations of Dicalcium Phosphate Dihydrate (DCPD) minerals, i.e., 0, 20, 30, 40 and 50 (wt)%. Increasing DCPD mineral concentration led to increased scaffold crystallinity, where the % crystallinity for CH, 20, 30, 40, and 50-DCPD scaffolds was determined to be 0.1, 20.6, 29.4, 38.8 and 69.9%, respectively. Reduction in scaffold pore size distributions was observed with increasing DCPD concentrations of 0 to 40 (wt)%; coalescence and close-ended pore formation were observed for 50-DCPD scaffolds. 50-DCPD scaffolds presented five times greater mechanical strength than the DCPD mineral-free scaffolds (CH). DCPD mineral enhanced cell proliferation for the 20, 30 and 40-DCPD scaffolds. 50-DCPD scaffolds presented reduced pore interconnectivity due to the coalescence of many pores in addition to the creation of closed-ended pores, which were found to hinder osteoblast cell proliferation.

## 1. Introduction

The metabolic and regenerative properties of bone are disrupted when the tissue is damaged from trauma and disease. The dynamic and highly vascularised bone tissue has a natural regenerative ability to self-repair small defects and cracks; however, when defects are critical-sized, i.e., >2.5 cm [1], the intervention of scaffolds is required. Bone is a highly specialised and complex living entity; therefore, potential bone scaffolds must express multiple properties collectively. An ideal bone scaffold should exhibit (i) osteoconductive potential to aid the formation of new bone; (ii) adequate mechanical strength to enable the scaffold to retain its structure during the implantation process, cell proliferation and also for load-bearing; (iii) appropriate microstructure to promote angiogenesis for nutrients circulation; (iv) resorbability to enhance void space to sure adequate room for bone cells to proliferate and differentiate; and (v) antibacterial properties [2]. Calcium phosphates (CaP) are bioceramics known to have a high affinity for bone morphogenetic proteins (BMPs), which encourage new bone tissue formation [3,4,5]. The capacity of CaP to form molecular interactions with surrounding tissues leading to surface apatite layer formation is referred to as osteoconductivity [6]. The rationale for the fabrication of CaP based bone scaffolds is related to the compositional similarity to natural bone minerals. CaP materials possess several advantageous properties, including biocompatibility, bioactivity, osteoconductivity and biodegradability [7]. CaP based ceramics have already gained approval for use in orthopaedic applications [8], including but not limited to cochlear implants, coatings for metal orthopaedic implants and bone fracture defect repairs.

The ability for bone scaffolds to resorb is essential to ensure space is created for new bone tissue to form and integrate into the implanted scaffold [8]. CaP ceramics have demonstrated: (i) predictable degradation rates, (ii) resorbability in vivo, and (iii) the progressive replacement of lamellar bone. Degradation refers to materials’ physical disintegration and fragmentation, whereas resorption refers to biodegradation via cellular mechanisms [9]. Thus, the process of resorption is classed as cell-mediated (phagocytosis by macrophages) and solution-driven [10,11]. The disintegration of CaP based materials causes particle formation, which leads to CaP resorption via phagocytosis by macrophages. The thermodynamical solubility of the CaP variations at pH 7 is **Dicalcium Phosphate Dihydrate** (DCPD) > **Octacalcium Phosphate** (OCP) > **Tricalcium Phosphate** (TCP) > **Hydroxyapatite** (HAP) from the most soluble to the least soluble [12]. Less stable phosphates such as DCPD, TCP and OCP precipitate biological apatite’s through intermediate steps and thus are considered precursors for bone mineralisation [13,14].

Potential bone scaffolds must have the capability to be fully resorbed once the bone has regenerated; the concept relies on the fundamentals of degradation where minerals/nanoparticles are released as the scaffold degrades over time to encourage an osteoconductive response. The degradation rate of the scaffold should match the regeneration rate of bone, and the scaffold must remain mechanically stable during bone cell proliferation. DCPD can be resorbed and is structurally stable at lower pH values (e.g., <5.5) compared to other CaPs [15]. DCPD is metastable under physiological conditions and converts to the more stable phases OCP and HAP [16,17,18,19]. DCPD is utilised as bone cement [20] due to being commonly found in pathological calcifications, i.e., mineralisation (osteoblast) in vitro [20,21]. Additionally, the biocompatible nature of DCPD has been demonstrated in many studies [22,23,24]; for example, new bone formation without inflammation is observed with the addition of DCPD in sheep cranial defect sites [25].

Despite the apparent beneficial properties exhibited by CaP materials, the low fracture strength and brittleness prevent CaP materials solely from being utilised in load-bearing applications [7]. Therefore, CaP minerals are commonly combined with other polymers (e.g., chitosan (CS)) to improve biological responses in terms of osteoblast response enhancement and direct mesenchymal stem cell phenotype, as well as to improve mechanical characteristics [1,7,26,27]. Chitosan (CS) is a copolymer consisting of β-(1→4) glycosidic linked D-glucosamine (deacetylated unit) and N-acetyl-D-glucosamine (acetylated unit) randomly distributed units [28,29,30]. CS exhibits a multitude of favourable properties, including non-toxicity, biodegradability [31,32], biocompatibility [31,33,34], antifungal, antibacterial [35] and wound healing abilities [36,37], thus is widely utilised in the biomedical, biotechnology and pharmaceutical fields [38]. Applications of CS include drug delivery, wound healing, bone scaffolds, cartilage and nerve tissue engineering [28,39]. CS is an ideal polymeric biomaterial able to be osteogenically and mechanically functionalised to fabricate potential bone scaffolds as it is easily functionalised due to the reactive primary amino and hydroxyl groups that allow side groups, peptides, and amino acids to bind [1,40]. The functional groups form stable covalent bonds during etherification and esterification reactions [41,42].

Previously, CS has been combined with osteogenic minerals, i.e., HAP [43,44]; the organic and inorganic material combinations have resulted in composites that can stimulate bone regeneration [31,40]. The proliferation of osteoblast cells improved for CS composites containing nano-HAP, which led to bone regeneration after eight weeks, as confirmed via micro-computed tomography [45]. Additionally, CS bone scaffolds have been demonstrated to support cell attachments and the proliferation of osteoblast cells, leading to in vitro mineralised bone matrix [1,46]. The incorporation of CaP minerals has also been shown to improve chitosan’s mechanical properties without conveying the disadvantage of pure mineral scaffolds’ propensity to fracture. The improvement in mechanical properties has been linked to CS reactive amino and hydroxyl groups, which are capable of crosslinking with materials containing at least two reactive functional groups, i.e., calcium phosphates, composites (nano-zirconia), nano-calcium zirconate), and bioglass [47]. Crosslinking bridges CS polymeric chains, leading to structural stabilisation [40]. The reduction in the CS protonated amino groups via crosslinking increases the mechanical properties of CS material, i.e., CS crosslinked with DiepoxyPEG (Diepoxy-polyethylene glycol) [40,48,49]. The compressive strength of freeze-dried CS scaffolds increases from 4 MPa to 11 Mpa for scaffolds containing CS- tricalcium phosphate [50].

This study aims to characterise and investigate the physicochemical properties of the porous chitosan scaffolds embedded with different concentrations of Dicalcium Phosphate Dihydrate (DCPD) (0, 20, 30, 40 and 50 wt %) fabricated using a freeze-drying approach.

## 2. Materials and Methods

*(1)*  *Dicalcium Phosphate Dihydrate Mineral (DCPD)*

The synthesis of DCPD mineral (CaHPO_4_•2H_2_O) was achieved via a slow drip wet precipitation route. Briefly, 200 mL of a 0.1 M Ca(NO_3_)_2_•4H_2_O (Fisher Chemicals, CAS: 13477-34-4, Hampton, VA, USA) aqueous solution (A) was heated to 37 °C. Then 200 mL of a 0.1 M (NH_4_)_2_ HPO_4_ (Acros Organics, CAS: 7783-28-0, Geel, Belgium) aqueous solution was added dropwise under continuous stirring to the solution (A). The resulting mixture was left stirring for 2 h at 37 °C. Then, the heat plate and the stirrer were switched off, and the mixture was left to settle for 1 h to allow precipitation (Equation (1)). The DCPD yield was filtered using Whatman Grade 44 filter paper (Merck, WHA1444110, Darmstadt, Germany) and washed three times using distilled water. The mineral collected was placed into a furnace and dried for 24 h at 80 °C.

Ca(NO_3_)_2_ · 4H_2_O + HPO_4_(NH_4_)_2_ → CaHPO_4_ · 2H_2_O + 2NH_4_NO_3_ + 2H_2_O (1)

*(2)*  *Chitosan (CS) Stock Solution*

The 3 (wt)% chitosan stock solution was prepared by dissolving high molecular weight chitosan flakes (Sigma-Aldrich, CAS: 9012-76-4, Taufkirchen, Germany, 3,100,000–3,750,000 Da, >75% deacetylated) in a 2 (*v*/*v*)% acetic acid (Acros Organics, Geel, Belgium, MFCD00036152) solution under continuous mixing for 24 h, after which the solution was placed into an ultrasonic water bath for 4 h for removal of air bubbles.

*(3)*  *Unloaded and DCPD Mineral Loaded Chitosan Scaffolds*

The scaffolds were fabricated by mixing different quantities of DCPD mineral, i.e., 20, 30, 40 and 50 (wt)%, to CS stock solutions under continuous stirring for 6 h. Measured amounts of unloaded and DCPD-loaded CS solutions were frozen at –80 °C for 24 h and then placed into a freeze drier (VirTis 4 KB ZL Benchtop K (SP Industries, Warminster, PA, USA)) set at −100 °C and pressure of 43 mTorr for 24 h.

*(4)*  *Alkaline Treatment*

The freeze-dried scaffolds were treated with 1 M sodium hydroxide (NaOH) (Sigma-Aldrich, CAS: 1310-73-2) for 10 min to reduce the dissolution rate of CS. The scaffolds were removed and blotted onto Whatman Grade 44 filter paper to remove excess NaOH residue. The treated scaffolds were then washed five times with distilled water to ensure traces of NaOH were removed. All synthesised freeze-dried scaffolds with corresponding sample code names are displayed in Table 1.

### 2.1. Characterisation Techniques

#### 2.1.1. Fourier Transform Infrared Spectroscopy (FTIR)

The molecular vibration spectroscopic analysis of the fabricated scaffolds was characterised using the attenuated total reflection (ATR) mode in the Vertex 70 FTIR spectrometer (Billerica, MA, USA). The beam splitter was KBr, and the light source used was a MIR lamp. Each scaffold was scanned 32 times in the 400 cm^−1^ to the 4000 cm^−1^ range at a spectral resolution was 4 cm^−1^.

#### 2.1.2. X-ray Diffraction (XRD)

A Bruker D8 X-ray diffractometer, Billerica, MA, USA, using the K_α_ radiation of Cu (λ = 0.15406 nm) was used to characterise the synthesised freeze-dried scaffolds. The scaffolds were analysed in the Bragg angle (2 θ) scanning range of 10° to 80° at a scan speed of 0.014° s^−1^ and step size of 0.065°. The recorded patterns were analysed using the HighScore Plus software (PANalytical X’Pert HighScore Plus v3.0, Malvern, UK), and the Rietveld refinement was employed for peak shape and intensity analysis for ascertaining the crystallinity of mineral samples.

#### 2.1.3. Scanning Electron Microscopy (SEM)

The morphology of the unloaded and DCPD mineral-loaded scaffolds was studied using the Hitachi SU8230 1–30 kV, (Düsseldorf, Germany) cold field emission gun SEM. Prior to SEM, the samples were coated with 6 µm of Iridium to improve the electrical conductivity of the materials, thus enabling an improvement with regards to signal-to-noise ratio. The SEM micrographs were processed and analysed using the ImageJ software version 1.41 USA, where the diameters of 60 random scaffold pores were averaged for each type of scaffold.

### 2.2. Testing Techniques

#### 2.2.1. Simultaneous Thermal Analysis (STA)

Thermal analysis of unloaded and DCPD mineral-loaded freeze-dried scaffolds investigated the decomposition of CS and the effect of mineral addition on the thermal degradation process. The Perkin Elmer STA 8000, Waltham, MA, USA was used to study the phase transformation and chemical reactions, covering the temperature heating range from 30 °C to 600 °C.

#### 2.2.2. Mechanical Testing

The Instron 5569 machine, USA was utilised to mechanically test rectangular freeze-dried scaffolds with 5 × 1 cm dimensions (*n* = 3). The scaffolds were sandwiched between pieces of polystyrene to prevent slipping and serve as an interface between the scaffolds and the tensile testing machine. The samples were tested with a 100 N load cell at a 100 mm/min strain rate with no pretension. Young’s modulus and ultimate tensile strength were determined from stress–strain plots.

#### 2.2.3. Scaffold Swelling

The scaffolds were dried at 50 °C for 5 h and weighed (*W_d_*) before the start of the experiment. The swelling characteristics of the freeze-dried scaffolds were determined by submerging the samples (*n* = 3) in phosphate buffer saline (Lonza, catalogue: BE17-517 Q, Basel, Switzerland) (PBS) solutions. The solution was distributed into individual glass beakers, and the scaffold samples were submerged at 37 °C for six h. After removing the samples from the PBS solutions at the specified times (0.5, 3 and 6 h), excess liquid was removed using Whatman Grade 44 filter paper. The samples were re-weighed using an electronic balance. The *swelling %* of the scaffolds was calculated using:

(2) $$\mathrm{Sweilling}\%=\left[\frac{W_{w}-W_{d}}{W_{d}}\right]\times 100$$

where *W_w_* and *W_d_* are wet and dry weights of the samples, respectively.

#### 2.2.4. Degradation Stability Testing

The physical integrity of the freeze-dried scaffolds was evaluated by soaking the samples (*n* = 3) in phosphate-buffered saline solutions. At scheduled time intervals, i.e., 1, 7, 14 and 28 days, the scaffolds were recovered and dried in a furnace oven at 50 °C for 24 h, then weighed. The weight loss percentages were calculated using Equation (3).

(3) $$\Delta W_{0}\left(\%\right)=\left[\frac{W_{0}-W_{d1}}{W_{d1}}\right]\times 100$$

where *W*_0_ and *W_d_*_1_ refer to the initial scaffold weights and the scaffold weights at time *(t)*, respectively.

#### 2.2.5. Zeta Potential

The zeta potential of unloaded and DCPD mineral-loaded CS suspensions was prepared by diluting the suspensions to concentrations of 2.9 g/dm^3^. The Melvern Zetasizer equipment was utilised, and the measurements were taken in cell DTS 1070 cuvettes. The refractive index of chitosan and DCPD minerals used were 1.52 and 1.65, respectively.

### 2.3. In Vitro Testing

The freeze-dried scaffolds, i.e., CH, 20, 30, 40 and 50-DCPD with dimensions of 1 cm diameter and 0.5 cm height, were sterilised with 70 (*v*/*v*)% ethanol for 10 min, washed five times using Dulbecco’s Phosphate Buffered Saline (DPBS), and then underwent UV light radiation for 1 h. Cell line G292 (sourced from the Department of Oral Biology Leeds University Dental School, purchased from ATCC, Manassas, VA, USA) were cultured in McCoy’s 5 A medium supplemented with 2 mM L-glutamine, 10% fetal bovine serum (FBS), and 100 units/mL penicillin with 100 ug/mL streptomycin (Sigma-Aldrich, Germany), and maintained at 37 °C with 5% CO_2_ in a humidified incubator. The cell media was replaced twice a week, and when the cells reached sub-confluence, they were passaged using 0.25 trypsine-0.1% EDTA.

#### 2.3.1. Contact Cytotoxicity Assay by Giemsa Staining

Scaffolds (*n* = 3) were attached to 6-well plates with the aid of steri-strips pieces (Medisave, cat no. R1540C); the positive and negative controls consisted of steri-strips pieces attached to the bottom of the wells and 40% dimethyl sulfoxide (DMSO), respectively. Dulbecco’s Phosphate Buffered Saline (DPBS) was used to wash the wells twice, aspirated, and 2 mL of G292 cell suspension containing 1 × 10^4^ cells were added to each well. The culture plates were incubated at 37 °C for 48 h in 5% CO_2_ in an incubator. After 48 h, the media was aspirated from the wells and washed twice with DPBS. 1 mL of 4 (*v*/*v*)% neutral-buffered formalin (NBF) was added to each well and incubated for 15 min. The formalin was aspirated, and all wells were stained for 5 min using Giemsa solution, then subsequently washed using distilled water. The culture plates were air-dried for 24 h and examined microscopically to record any changes in morphology, confluency, attachment, and detachment of the G292 cells using the Leica CTR HS microscope under bright field illumination. All images were collected digitally.

#### 2.3.2. Fluorescence Actin and Nuclei Staining

For cellular adhesion identification, 2 × 10^4^ cell line G292 cells were seeded onto scaffolds and left for 18 h to adhere to the scaffold surfaces. Cell seeded scaffolds were washed twice with PBS, then fixed with 1% neutral-buffered formalin (NBF), permeabilised with 1 (*v*/*v*)% Triton X-100 (Sigma Aldrich, Germany) for 5 min and then washed twice using PBS. The cell-seeded scaffolds were incubated with Alexa Flour-488 phalloidin (Invitrogen, Waltham, MA, USA) for 2 h to stain the actin filaments and co-incubated with 4′,6-diamidino-2-phenylindole DAPI dye (Sigma-Aldrich) for 15 min to stain the cell nuclei. Stained cells were rinsed with PBS twice and then imaged using Leica TCS SP8 confocal microscope (Germany).

#### 2.3.3. Extract Cytotoxicity by XTT Assay

The scaffold eluates were prepared according to the ISO standard: ISO10993-12:2007 part 12. Briefly, scaffolds in triplicate were placed into 24 well plates containing 2 mL of supplemented McCoy’s 5 A media and incubated at 37 °C in 5% CO_2_ for 1, 3, and 7 days. At each time point, 330 µL aliquots (*n* = 6) of the media were collected and frozen in cryovials at −80 °C until required. Extract cytotoxicity evaluation of the synthesised freeze-dried scaffolds was assessed according to the ISO10993-5:2009(E) part 5: Tests for in vitro cytotoxicity. Briefly, cell line G292 osteoblast cells were seeded into 96-well plates at a cell density of 5000 cells/well and incubated at 37 °C in 5% CO_2_ for 24 h. After the stipulated time, the media was aspirated from the wells and replaced with 100 µL of the thawed collected media containing scaffold eluates. The negative and positive controls consisted of McCoy’s 5 A media with 10% DMSO and McCoy’s 5 A media, respectively. The well plates were incubated at 37 °C in 5% CO_2_ for 24 h. After 24 h, the media in the wells were removed and replaced with 100 µL of McCoy’s 5 A media, 10% FCS and 50 µL of the XTT assay solution, then incubated for 4 h at 37 °C in 5% CO_2_. After the stipulated time, 100 µL were aliquoted into new 96 well plates and read on a microplate reader at 450, 570 nm and 630, 670 nm (reference wavelengths). The values at 650 nm were deducted from 450 nm to obtain the final optical density (OD). The test well ODs were normalised to the positive control ODs to measure cell viability.

#### 2.3.4. DNA Quantification by Picogreen Assay

Scaffolds were seeded with G292 osteoblast cell suspension containing 10^4^ cells and incubated for 1, 3 and 7 days. The media was changed every two days. Cell proliferation examined using Quant-iT™ PicoGreen™ dsDNA Assay Kit (ThermoFisher Scientific, Waltham, MA, USA, CAT: P7589) was performed according to the manufacturer’s instructions. Briefly, the samples were washed three times with DPBS, and lysis of the cells was carried out using a lysis solution (0.2% (*v*/*v*) Triton x-100, 10 mM Tris-HCl, 1 mM EDTA, pH 7.4) for 1 h on a gyratory shaker. 100 µL of the PicoGreen dsDNA quantification reagent was diluted to 1:200 and added to a 96-well plate containing 100 uL of the cell lysate solutions. The DNA standard curve prepared via serial dilutions of the Lamda DNA standard in TE buffer (10 mM Tris-HCl, 1 mM EDTA, pH 7.4) was added to the 96-well plate instead of the sample lysis solutions in addition to 100 uL of the PicoGreen solution. The 96-well plate was incubated in the dark for 5 min before the absorbance was measured using a Thermo Scientific Varioskan Flash plate reader, excitation at 480 nm and emission at 520 nm. All the data collected were calibrated using a DNA standard curve.

### 2.4. Statistical Analysis

Data are presented as mean ± standard deviation. Analysis of the differences between groups was performed using two-way ANOVA. Statistical analysis and graphical representations of the data were implemented using GraphPad Prism (version 9.2.0, USA). The results were considered significant at a *p*-value of < 0.05.

## 3. Results

### 3.1. Fourier Transform Infrared Spectroscopy

**Figure 1:**
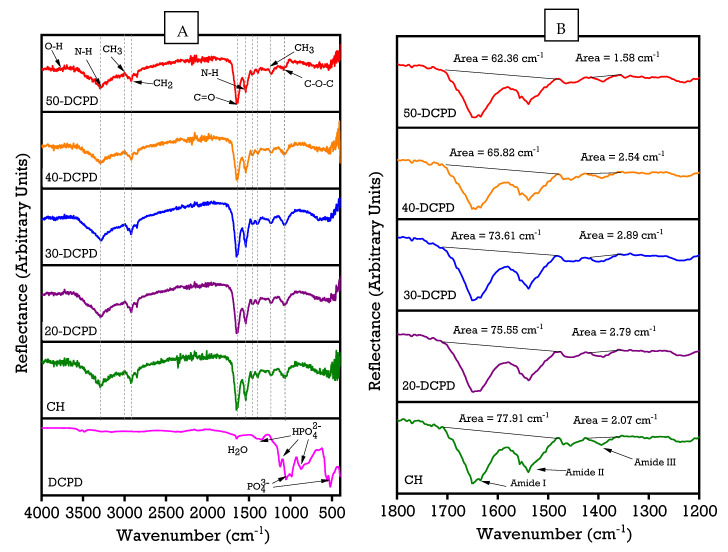
Comparison of Fourier transform infrared spectroscopy spectra of synthesised freeze-dried chitosan (CS) scaffolds containing varying concentrations of dicalcium phosphate dihydrate (DCPD) mineral (0, 20, 30, 40 and 50 (wt)% DCPD): (**A**) data obtained in the 400 cm^−1^ to 4000 cm^−1^ regions at a resolution of 4 cm^−1^ using the Vertex 70 FTIR spectrometer, USA, in attenuated total reflection mode; (**B**) comparison of the amide I, II and III peaks determined using OriginPro software.

The FTIR spectra of synthesised unloaded and DCPD mineral-loaded freeze-dried scaffolds are presented in Figure 1. The FTIR bands associated with DCPD mineral are located at 511 cm^−1^, 989 cm^−1,^ and 1075 cm^−1^, which relate to PO_4_^3-^ contributions. The bands located at 857 cm^−1^, 1117 cm^−1,^ and 1217 cm^−1^ correspond to the contributions of HPO_4_^2−^. The freeze-dried CH scaffold presents broad transmission bands in the 3290 cm^−1^ and 2993 cm^−1^ regions, attributed to N-H and O-H bond stretching of the saccharide ring, respectively. The peak at 2913 cm^−1^ relates to CH_2_ symmetric/asymmetric pyranose ring vibrations. The amide stretching vibration C=O (amide I) depicted at 1625 cm^−1^ is directly related to the backbone conformation. The N-H bending vibration (amide II) is displayed at 1541 cm^−1^, and the C-N stretching vibration (amide III) corresponds to 1396 cm^−1^. The peak at 1457 cm^−1^ relates to the CH_3_ deformation mode [51]. In contrast, the 1059 cm^−1^ region correlates to C-O-C stretching vibration, which depends on the crystallinity of chitosan. The saccharide structure of chitosan also exhibits a general reflection associated with the >846 cm^−1^ regions. The observations agree with previously reported data [52,53,54,55]. The DCPD mineral-loaded freeze-dried scaffolds present similar FTIR spectra to the unloaded CH scaffold; this may be attributed to the CS amide (I, II and III), CH_3_ and saccharide bands overlapping the HPO_4_^2−^ and PO_4_^3−^ peaks associated with DCPD mineral. The literature suggests that the CS protonated amino groups (NH_3_^+^) can form strong intermolecular interactions with the DCPD mineral phosphate groups (divalent HPO_4_^2−^, trivalent PO_4_^3^HPO_4_^2−^) [56,57]. Figure 1B displays the calculated areas of the amide I, II and III peaks, where a clear decreasing area trend is observed with increasing DCPD mineral concentrations. As the concentration of DCPD mineral increases, the number of divalent HPO_4_^2^PO_4_^3^HPO_4_^2−^ and trivalent PO_4_^3−^ groups also increases, leading to a greater probability of interactions with protonated CS amino groups forming ionic crosslinking, leading to the reduction in the calculated areas [58].

### 3.2. X-ray Diffraction

**Figure 2:**
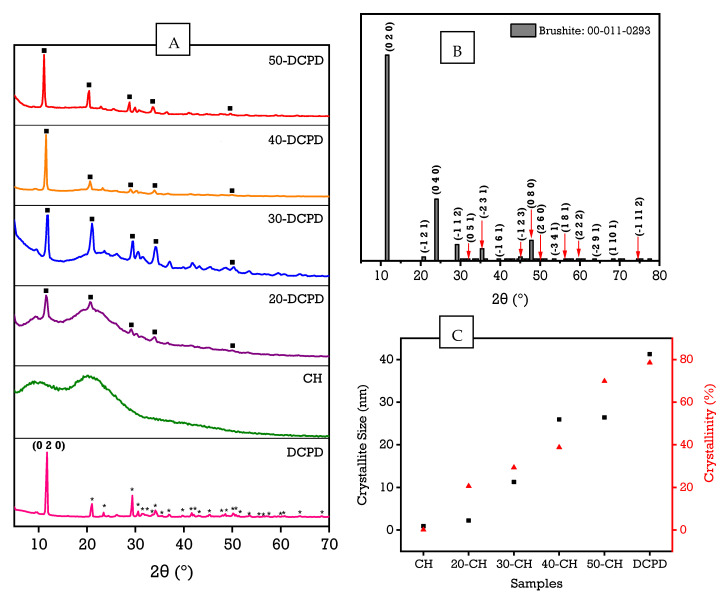
Normalised X-ray diffraction data, (**A**) Experimental XRD spectra for DCPD, CH, 20, 30, 40 and 50-DCPD samples, (**B**) DCPD reference spectra and (**C**) graph depicting the relationship between crystallite size and crystallinity. * corresponds to Bragg 2θ diffraction peaks of DCPD while ▪ corresponds to miller indices (0 2 0), (0 4 0), (−1 1 2), (−2 3 1) and (0 8 0) diffraction planes, respectively.

The experimental XRD diffraction patterns for the DCPD, CH, 20, 30, 40 and 50-DCPD synthesised scaffolds are presented in Figure 2. The peaks from the obtained DCPD pattern match the reference standard XRD data for DCPD (JCPDS: 00-011-0293) compiled by the Joint Committee on Powder Diffraction and Standards (JCPDS). The main 2 θ peaks for the DCPD standard are 11.60°, 23.39°, 29.16°, 35.45° and 47.84° corresponding to (0 2 0), (0 4 0), (−1 1 2), (−2 3 1) and (0 8 0), respectively. Notably, Dicalcium Phosphate Dihydrate is the only detectable phase expressed in the DCPD sample. The partially crystalline polysaccharide CS has a characteristic XRD fingerprint associated with two broad 2θ peaks at ~10° and ~20°, corresponding to crystal I and II phase forms, respectively [59]. The less hydrated crystal I phase exhibits higher crystallinity, while the crystal II phase has a hydrated amorphous structure corresponding to intermolecular interactions of the aligned CS polymer chains. For the CH freeze-dried scaffold, two broad peaks at 9.2° and 20.2° are observed, and the lack of other peaks in the CH diffraction pattern signifies high CS purity. Increasing DCPD concentration caused a significant decrease in the CS crystal II phase form for the freeze-dried scaffolds containing DCPD mineral, which is expected due to the high crystallinity of DCPD mineral. The diffraction patterns for 20, 30, 40 and 50-DCPD depict evidence for the semi-crystalline CS matrix combined with the characteristic crystalline diffraction peaks associated with DCPD. As DCPD concentration increases, the partially crystalline CS structure becomes more crystalline, where the 50-DCPD diffraction pattern is similar to the DCPD spectra, as shown in Figure 2A.

Rietveld Refinement was performed, and the crystallinity % of the scaffolds was calculated by subtracting the area of crystalline peaks from the total area of all peaks. The overall crystallinities of the freeze-dried scaffolds are displayed in Table 2. The crystallite size range for the synthesised scaffolds was determined from X-ray line broadening data using Scherrer’s equation B_D_ = Kλ/D cos (θ), where λ refers to the incident instrument Cu k_α1_ radiation wavelength, D corresponds to the crystalline size, and K is a shape factor constant ~0.9. The crystallite size is independent of crystallinity; however, based on the results displayed in Figure 2C, both the crystallite size and crystallinity increase with increasing DCPD mineral concentration.

### 3.3. Scanning Electron Microscopy

**Figure 3:**
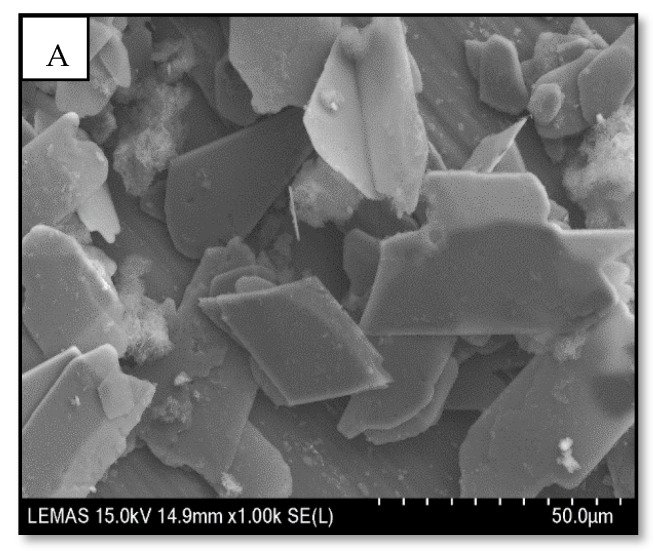

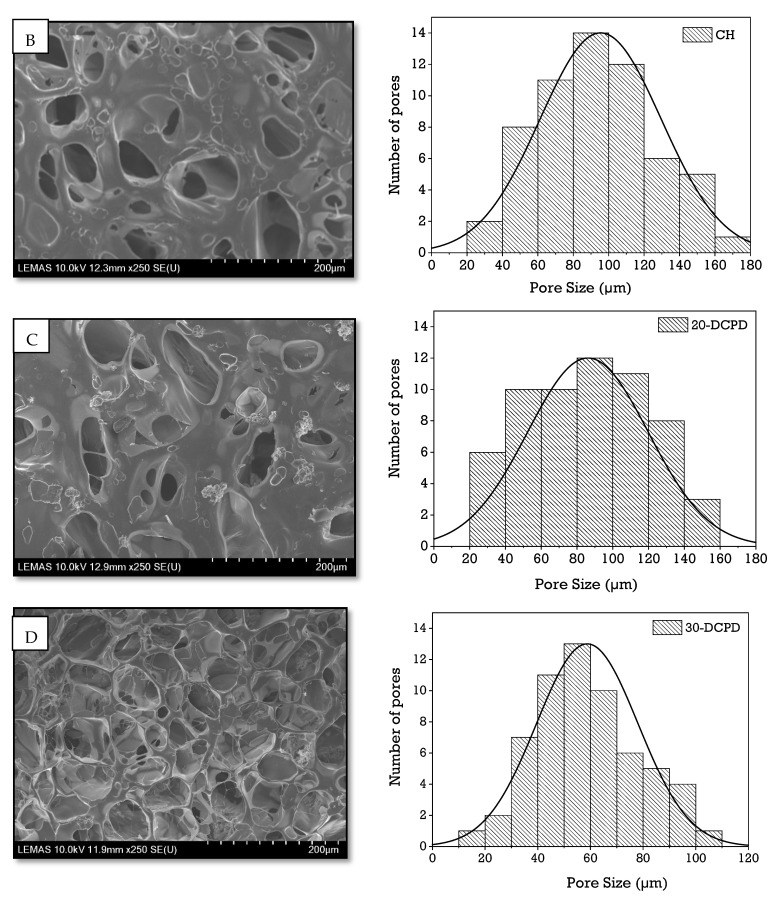

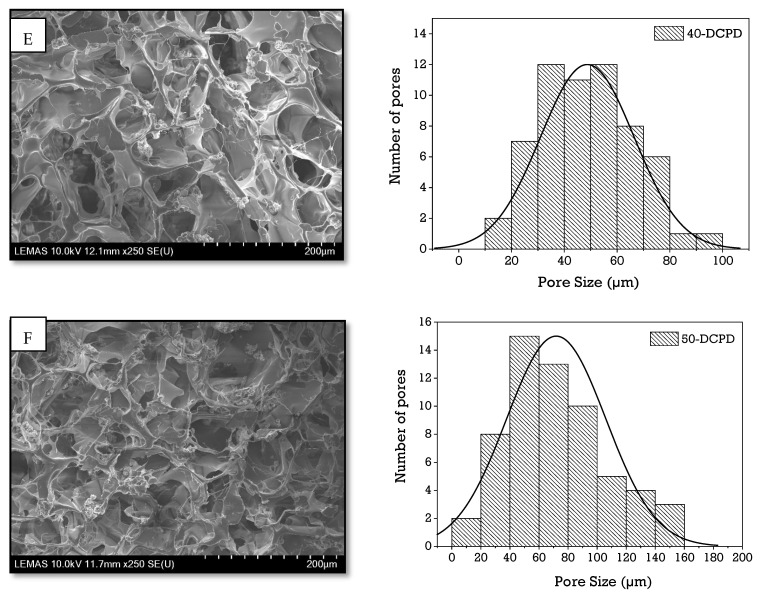
Comparison of Hitachi SU8230 SEM images of freeze-dried chitosan (CS) scaffolds containing Dicalcium Phosphate Dihydrate (DCPD) minerals (**A**) DCPD mineral, (**B**) CH, (**C**) 20-DCPD, (**D**) 30-DCPD, (**E**) 40-DCPD, and (**F**) 50-DCPD. The corresponding distribution graphs.

Figure 3 displays the SEM images of the porous architecture of freeze-dried scaffolds fabricated using various concentrations of DCPD mineral (20, 30, 40 and 50 (wt)%). As depicted, increasing the DCPD mineral content affected the morphology and structural features of the scaffolds. The CH scaffold containing no DCPD mineral displays relatively thick lamellae with a broad pore size distribution; the pore sizes range from 20 µm to 180 µm. The incorporation of DCPD mineral caused the pore size distribution and the lamellae thickness to decrease while pores increased. The structural difference and increase in the number of pores are especially visible compared to the 20 and 30-DCPD scaffolds. The 30-DCPD scaffolds exhibit >70% more pores when compared to the 20-DCPD scaffold, with the majority of pores ranging from 20 µm to 160 µm. As the DCPD mineral concentration increased to ≥40 (wt)%, the shape of the pores became less defined, and the lamellae thickness further decreased, as demonstrated in Figure 3E. The reduction in lamellae sizes consequently caused many pores to combine; hence individual pores are no longer visible, as depicted in Figure 3F for the 50-DCPD scaffold.

### 3.4. Simultaneous Thermal Analysis

**Figure 4:**
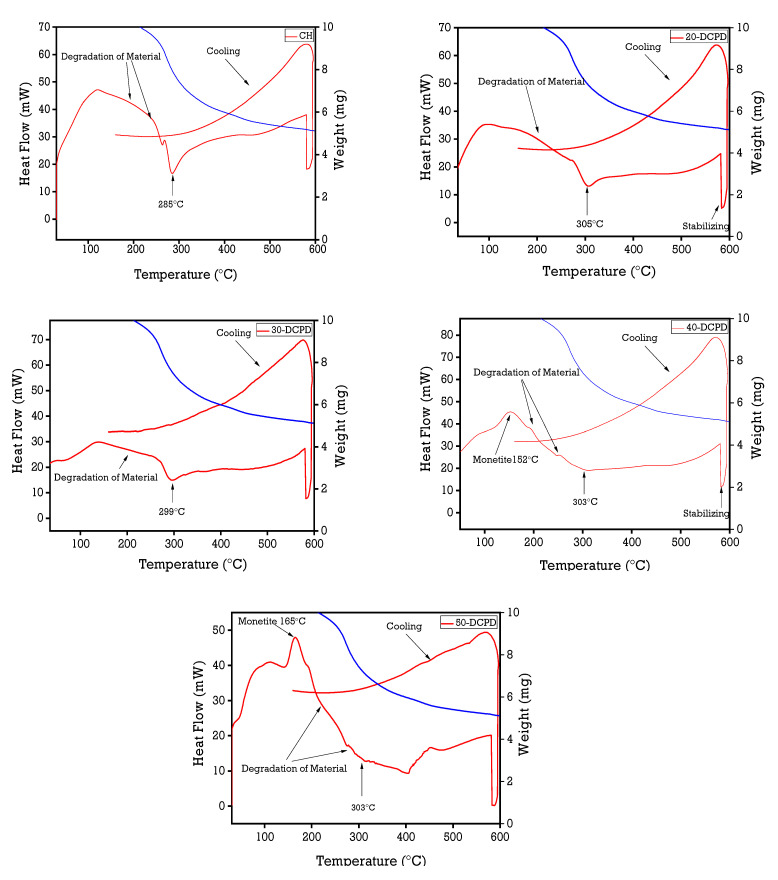
Thermal analysis of high molecular weight freeze-dried chitosan scaffolds using the Perkin Elmer STA 8000 from 30 to 600 °C at a heating and cooling rate of 20 °C/min. The red line refers to the heat flow, while the blue line refers to the mass change of the sample.

Figure 4 displays the thermal analysis data of all synthesised freeze-dried scaffolds. The observed initial temperature weight loss below 100 °C is attributed to the samples’ evaporation of moisture (water) from hydrophilic groups. The shoulder at approximately 120 °C expressed in all samples corresponds to the loss of adsorbed and bound water [60,61,62]. The CH, 20 and 30-DCPD scaffolds present similar downward slopes from 100 °C to 160 °C, representing the beginning of the thermal degradation of CS, and the second weight loss observed at 160 °C to ~300 °C is attributed to the decomposition of the CS biopolymer chains [62].

High molecular weight (M_w_) CS is thermally stable [63,64] due to the extensive amount of hydrogen bonding compared with low/intermediate M_w_ CS. Therefore, the increase in weight loss for the DCPD mineral-loaded scaffolds is likely attributed to the increasing presence of phosphate ions, forming electrostatic interactions with the protonated CS amino groups, thus leading to more significant thermal degradation. Monetite, also known as Dicalcium Phosphate Anhydrous, is formed via the dehydration of DCPD. Therefore, the endothermic peaks observed for the 40 (152 °C) and 50-DCPD (165 °C) scaffolds are attributed to the transformation of DCPD mineral to monetite (Equation (4)).

2CaHPO_4_ 2H_2_O (DCPD) → 4H_2_O + 2CaHPO_4_ (Monetite) (4)

### 3.5. Mechanical Properties

Increasing DCPD mineral concentrations improved Young’s modulus and tensile strength for freeze-dried scaffolds containing DCPD minerals, as displayed in Table 3. The most significant modulus and strength enhancement are exhibited for the 30-DCPD scaffolds compared with the 20-DCPD scaffolds, where Young’s modulus and tensile strength increased by 65.29% and 63.26%, respectively. Incorporating DCPD minerals into the freeze-dried chitosan scaffolds increased crystallinity, as confirmed via XRD analysis (Figure 2), thus stabilising and restricting the CS biopolymer chains. The scaffolds containing increased DCPD minerals required greater forces to pull part of the chitosan biopolymer chains, therefore corresponding to enhanced mechanical properties overall.

### 3.6. Scaffold Swelling and Degradation

**Figure 5:**
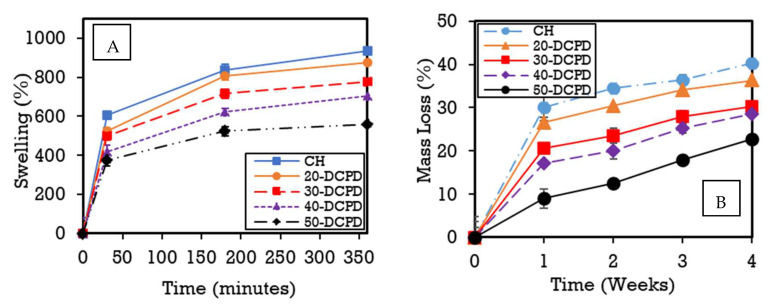
(**A**) Swelling kinetics of CH, 20, 30, 40 and 50-DCPD freeze-dried scaffolds submerged in phosphate saline buffer (pH 7.4) at a physiological temperature of 37 °C. Experiments were carried out in triplicates. (**B**) Degradation results for CH, 20, 30, 40 and 50-DCPD freeze-dried scaffolds submerged in phosphate saline buffer (pH 7.4) at a physiological temperature of 37 °C. The experiments were carried out in triplicate over a 4-week process.

The swelling behaviour of the freeze-dried CS scaffolds containing various concentrations of DCPD mineral at different time intervals is displayed in Figure 5A. The patterns observed indicate an initial rapid swelling increase from 0 to 25 min for all the scaffolds. There is a collective gradual increase between 25 and 175 min, followed by mass stabilisation from 175 to 350 min. The results reveal that the DCPD mineral concentrations strongly influenced the swelling % as it varies from 934.7 ± 23.3% for un-loaded DCPD mineral CH freeze-dried scaffolds to 557.4 ± 29.8% for 50-DCPD mineral freeze-dried scaffolds. The equilibrium swelling % is lower for scaffolds containing DCPD minerals. The swelling % experiments confirm that all the polymer matrixes of the synthesised scaffolds can swell and store water similarly to living tissues [35]. The swelling % decreased with increased DCPD mineral content. The degradation behaviour of the synthesised freeze-dried scaffolds is displayed in Figure 5B. Increasing DCPD mineral concentration promoted a reduction in the mass loss of the scaffolds, whereby 50-DCPD scaffolds exhibited the lowest mass loss of 22.7 ± 1.2% compared to CH freeze-dried scaffolds, which expressed 40.3 ± 1.7% mass loss. The 20, 30, and 40-DCPD scaffolds presented mass losses of 36.3 ± 1.5%, 30.2 ± 1.3% and 28.6 ± 1.0%, respectively, at four weeks. Scaffolds that exhibited higher crystallinity, i.e., 40 and 50-DCPD, as investigated using XRD analysis displayed in Figure 2, expressed lower equilibrium swelling and degradation degrees.

### 3.7. Zeta Potential

The zeta potential, i.e., ±30 mV, is considered stable for colloidal systems due to the surface charge particle repulsions. The agglomeration tendency is conveyed by the Derjaguin Landau Verwey Overbeek (DLVO) theory, which corresponds to the sum of the electrostatic repulsive and Van Der Waals forces, thus, determining the total interaction energy at a particular separation distance [65]. The zeta potential of DCPD mineral is −12.44 ± 0.4 mV, indicating the agglomeration potential [66,67]. Agglomeration of the synthesised DCPD mineral is also confirmed from the SEM characterisation shown in Figure 3A. The negative surface charge density value for DCPD is related to the presence of phosphate groups (PO_4_^3−^) within the structure. Conversely, the zeta potential values for all freeze-dried scaffolds are positive, confirming the protonation of the amino groups, which gave rise to the overall positive charge. However, the charge decreases with increasing DCPD mineral concentration, where the average zeta potential decreases (Table 4) from ±43.5 ± 0.4 mV to ± 20.2 ± 0.5 mV for DCPD free and 50-DCPD mineral loaded freeze-dried scaffolds, respectively. The interaction of DCPD phosphate groups (PO_4_^3−^) with the protonated CS scaffold amino groups (−NH_3_^+^) is likely attributed to the decrease in overall positive charge exhibited by the scaffolds.

### 3.8. Extract Cytotoxicity by XTT Assay and DNA Quantification by Picogreen Assay

**Figure 6:**
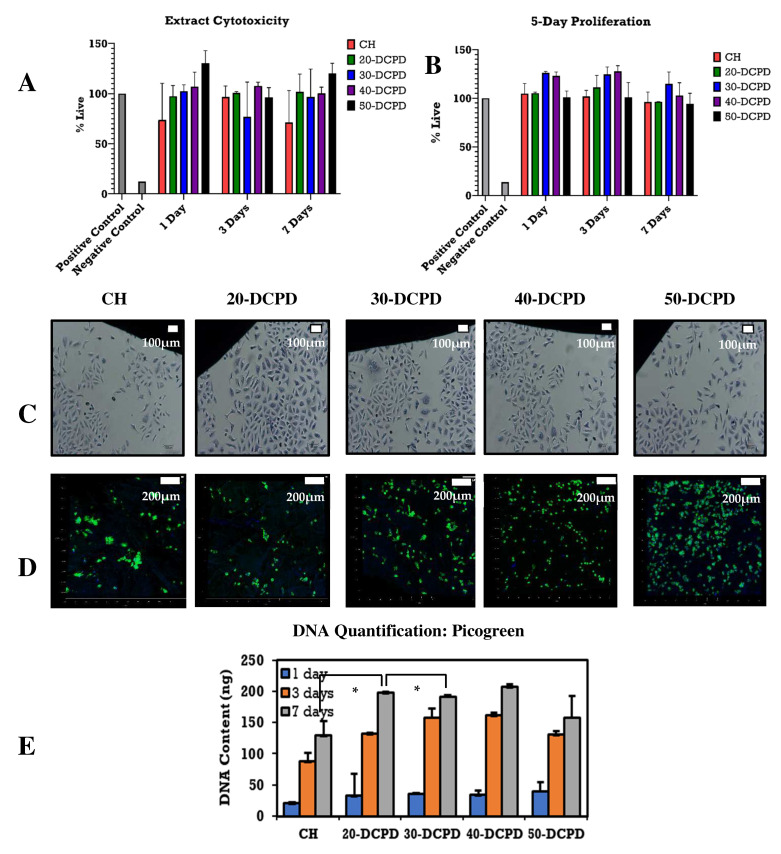
Extract cytotoxicity and proliferation testing using osteoblast cell line G292 cells on freeze-dried scaffolds. Positive and negative controls consisted of steri-strips and McCoy’s media with 40% DMSO. (**A**) Extract cytotoxicity results where testing consisted of 5000 cells/well G292 osteoblast cells, measured as the percentage of extracts collected after 1, 3 and 7 days. (**B**) 5-day cell proliferation percentage live extract cytotoxicity results where testing consisted of 500 cells/well G292 osteoblast cells. (**C**) Contact cytotoxicity testing of Giemsa-stained cell line G292 osteoblast cells for unloaded and DCPD mineral-loaded freeze-dried chitosan scaffolds (objective ×20). Images were collected digitally using the Leica CTR HS microscope under bright field illumination. All samples were compared to negative (steri-strips) and positive (40% dimethyl sulfoxide (DMSO)) controls. (**D**) Fluorescence microscopy images of cell line G292 osteoblast cells adhered to scaffold surfaces. After 48 h of cell seeding, samples were fixed and processed using Alexa Fluor-488 phalloidin to label actin (green) and Dapi to label nucleic acids in the nuclei (blue) (objective ×10). (**E**) Cell proliferation of undoped and mineral-doped freeze-dried scaffold evaluated using PicoGreen assay. The error bars are equivalent to the mean ± standard deviation (SD) (*n* = 3 in each group). The * refers to statistical significance when *p* < 0.05.

The indirect extract cytotoxicity assay results (Figure 6A) indicate ~70% cell viability at all time points, i.e., 1, 3 and 7 days. Nearly all freeze-dried scaffold types presented increased ODs compared to the positive controls, especially the scaffolds containing increased concentrations of DCPD minerals, i.e., 50-DCPD. The cell proliferation measured seems to increase with increasing DCPD mineral concentrations, as confirmed by the 5-day proliferation results depicted in Figure 6B. Cell proliferation for all the freeze-dried scaffolds is more significant than positive controls, indicating that DCPD minerals do not hinder cellular growth. Secondly, DCPD minerals also appear to aid cell proliferation. The quantitative Picogreen assay used to assess cellular viability of freeze-dried scaffolds, i.e., CH, 20, 30, 40 and 50-DCPD, confirmed successful cell proliferation at 1, 3 and 7 days, as shown in Figure 6E. The general trend for the CH, 20, 30 and 40-DCPD at each time point presents an increase in the proliferation of cells. For the 50-DCPD scaffold, a reduction in osteoblast proliferation is observed at 3 and 7 days compared to 40-DCPD.

### 3.9. Contact Cytotoxicity and Fluorescence Staining

The in vitro cytotoxic effects of the freeze-dried scaffolds were evaluated qualitatively, as depicted in Figure 6C. Microscopic examinations of the osteoblast cells proliferated on or near the samples in terms of general morphology, membrane integrity, and cell attachment shows little or no cytotoxic effect. The negative control of DMSO resulted in a lack of cell growth due to cell lysis. The freeze-dried samples present similar results to the positive control due to osteoblast cells successfully proliferating entirely around each scaffold. The morphology of the osteoblast cells is consistent throughout all the freeze-dried scaffold samples, i.e., CH, 20, 30, 40 and 50-DCPD, and the lack of cell lysis indicates the biocompatible nature of the materials. Interactions of the freeze-dried scaffolds with G292 osteoblast cells were assessed by observation of the attachment, cell morphology and survival of cells in vitro. Confocal images (Figure 6D) of the cell-seeded scaffolds stained with Alexa Flour-488 phalloidin and DAPI after 24 h revealed metabolically active cells distributed across the surface of the scaffolds where increasing DCPD concentrations led to an increase in cell proliferation.

## 4. Discussion

Bone scaffold porosity and pore interconnectivity are essential for the adhesion, proliferation, differentiation of bone cells, transporting nutrients, and waste removal [68,69]. Adequate porosity aids revascularisation [70] when scaffolds are implanted in vivo [71]. Within the tissue engineering industry, the mean pore size for bone scaffolds has been investigated to be between 50 µm and 1500 µm [72,73], with a minimum pore size range of 50 µm to 100 µm required to exhibit adequate bone and tissue regeneration [69,74,75]. As confirmed via SEM analysis (Figure 3), the bone scaffolds fabricated via the freeze-drying approach exhibited highly interconnected porous structures with variations in pore size distributions. The pore size distribution and amount of porosity are affected by the initial freezing temperatures of the scaffolds. Freezing temperatures, i.e., >−60 °C, increase the cooling rate creating a lower freezing temperature of the CS suspensions, which induces a greater driving force for pore nucleation. Thus, the resulting structures contain a higher number of smaller pore sizes [76]. The CH scaffold expressed the most extensive pore size distribution (20 to 180 µm); however, increasing DCPD mineral concentrations led to a significant increase in the number of pores and a reduction in the pore size distributions, i.e., 10 µm to 160 µm (20-DCPD), 10 to 110 µm (30-DCPD), 10 to 100 µm (40-DCPD) and 10 to 160 µm (50-DCPD). Smaller pore size distributions enhance the surface areas of scaffolds by providing increased sites for cellular attachment. Pore sizes <50 µm have been found to limit cell migration, form cellular capsules, and in severe cases, lead to necrotic regions as the diffusion of nutrients and waste is restricted [77,78]. Large pores >1500 µm lead to a reduction in the scaffold surface area, which is found to limit the adhesion of cells [79]. Hence, pore size must be large enough to allow for the migration of cells throughout the scaffold and small enough to allow cell binding to the scaffold [73,80].

The number of pores associated with the 50-DCPD scaffold reduced significantly. The majority of pores coalesced, forming less defined microstructures with reduced pore interconnectivity, which is unfavourable for cell growth as confirmed via the DNA quantification analysis results (Figure 6C), where a reduction in G292 cell proliferation is observed on days three and seven as compared with the CH, 20, 30 and 40-DCPD freeze-dried scaffolds. The coalesces, and the formation of closed-ended pores likely reduced the flow of nutrients, thus reducing G292 osteoblast proliferation. Potential bone scaffolds must have the ability to absorb inflammation liquids during wound healing in a timely manner to avoid infection of the wound [81]. Adequate liquid absorption capability has been found to promote cell adhesion but lower mechanical properties of scaffolds [35,62,82]. Additionally, the ability of freeze-dried scaffolds to swell and retain a certain amount of water/liquid within their structure is essential for controlled release applications, i.e., drugs or minerals [83]. CS is a hydrophilic biopolymer [84] that facilitates the diffusion of water molecules due to the structural free volume and the ease of polymer chain mobility [85]. Therefore, the DCPD mineral-free CH scaffolds presented the highest liquid uptake, while the 50-DCPD scaffolds exhibited the lowest swelling % increase. The equilibrium swelling % (Figure 5A) is lower for scaffolds containing DCPD mineral and is likely to be attributed to the reduction in the hydrophilic functional groups in the cationic CS structure, i.e., amine (NH_2_) and amide (-CONH, -CONH_2_) groups [86], due to the interaction of the divalent HPO_4_^2−^ and trivalent groups PO_4_^3−^ from DCPD minerals. The hydrophilic groups are water-binding sites [86,87], resulting in the expansion and occupation of a larger volume. Reducing the number of hydrophilic groups in the CS structure is unfavourable to the swelling rate [88]. Thus, increasing DCPD concentrations restricted the mobility of the CS biopolymer chains, reducing the water molecules’ movement capability into the scaffolds. The swelling % results confirm that all the polymer matrixes of the synthesised scaffolds can swell and store liquid, which is favourable for living tissues [35].

Potential bone scaffolds and the scaffold degradation products must be biocompatible to ensure no cytotoxicity or inflammatory response is induced when implanted in vivo [89]. The surrounding tissues may not eliminate acid by-products, leading to either a toxic or inflammatory response. Conversely, the growth of new bone could be impeded if the degradation rate of the bone scaffold is too slow [76]. Therefore, potential bone scaffolds should be tuned to degrade at a similar rate to the regeneration rate of bone to ensure that the degraded scaffold material is replaced by the regenerated bone tissue [1]. The rate of bone regeneration depends on the fracture size and can range from 50 to 100 µm/day for contact healing [90] and 3 to 8 weeks for gap healing. The synthesised scaffold degradation results (Figure 5B) indicate increasing the DCPD mineral concentration reduces the scaffold mass loss. The mass loss reduction is related to the degree of deacetylation (DD), molecular weight (M_w_) and crystallinity, as confirmed by other researchers [91]. The freeze-dried CH scaffolds presented the most significant mass loss of 40.3 ± 1.7%, while the 50-DCPD scaffolds exhibited the lowest mass loss at 22.7 ± 1.2% after four weeks. The difference is likely attributed to the 50-DCPD exhibiting increased crystallinity, as confirmed from the XRD analysis compared to the CH, 20, 30 and 40-DCPD scaffolds. Increased crystallinity leads to extensive hydrogen bonding and intermolecular forces between the CS biopolymer chains, resulting in a more compact scaffold structure, thus reducing the water molecule’s accessibility to the hydrophilic groups and reducing the degradation rate.

The zeta potential of CS is dependent upon the M_w_. The structure of high M_w_ CS consists of longer polymeric chains indicating increased functional groups compared to low M_w_ CS. Therefore, the relative positive charge (+ve) corresponds to the number of protonated amino groups in the CS structure. As expected, the DCPD mineral-free DCPD scaffolds presented the highest zeta potential value of +43.47 ± 0.35, while the zeta potential for DCPD mineral was −12.44 ± 0.4 mV. The zeta potentials of the synthesised freeze-dried scaffolds follow a decreasing trend whereby increasing DCPD concentration caused a reduction in the positive zeta potential values. The zeta potential reduction is correlated to increasing DCPD phosphate ions, forming ionic bonds or electrostatic interactions with the protonated amino groups in CS. Other researchers report similar findings [92,93].

Potential bone scaffolds must possess adequate mechanical strength to support cellular growth and maintain structural integrity during and after placement at the defect site [1]. CS alone does not possess the mechanical strength required for load-bearing applications. Thus, incorporating DCPD minerals with CS enhanced mechanical properties where the overall strength of the synthesised scaffolds increased with increasing DCPD mineral concentration. The 50-DCPD scaffolds expressed a 20.1 ± 0.54 kNm^−2^ increase in Young’s Modulus and a 20.1 ± 0.28 kPa increase in tensile strength compared to the DCPD mineral-free CH scaffolds. The improvement of mechanical strength is likely attributed to the restriction of the CS biopolymer chains by the HPO_4_^2−^ and PO_4_^3−^ ions associated with DCPD minerals. 50-DCPD scaffolds exhibited a reduction in the total porosity but presented the highest mechanical properties compared to other freeze-dried scaffolds, i.e., CH, 20, 30 and 40-DCPD. The reduction in total porosity and the subsequent increase in mechanical properties are likely attributed to the decrease in the total void volume.

## 5. Conclusions

The fabrication of porous freeze-dried CS scaffolds embedded with different concentrations of DCPD minerals (0, 20, 30, 40 and 50 (wt)%) was successful, as confirmed by the XRD, FTIR and SEM characterisation results. Increasing the DCPD mineral concentration from 0 to 50 (wt)% led to increased crystallinity of the scaffolds, which reduced the scaffold’s rate of degradation when immersed at 37 °C. The enhanced crystallinity with increasing DCPD mineral content provided further hydrogen bonding and intermolecular forces, thus restricting CS biopolymer chains reducing the overall scaffold’s liquid uptake. Incorporating DCPD minerals improved the mechanical properties, where 50-DCPD scaffolds presented five times greater mechanical strength than the DCPD mineral-free scaffolds (CH). The pore size distributions decreased with increasing DCPD mineral concentration (20 to 40 (wt)%). However, for scaffolds containing 50 (wt)% DCPD the porosity reduced as many pores coalesced, forming closed-ended pores. Since porosity and pore size plays an essential role in terms of osteoblast proliferation and differentiation, the negative effect of the reduced porosity is observed for the 50-DCPD scaffolds in terms of cellular growth. Increasing DCPD concentration led to increased osteoblast proliferation for the 20, 30 and 40-DCPD scaffolds. However, osteoblast reduction was observed at days three and seven for the 50-DCPD scaffolds compared with the 40-DCPD scaffolds. The reduction is attributed to the change in the scaffold architecture and reduced porosity. Overall, the incorporation of DCPD minerals with chitosan enhanced mechanical and osteogenic properties.

## Figures and Tables

**Table 1:** The synthesised Dicalcium Phosphate Dihydrate mineral and freeze-dried scaffolds with corresponding code names.

Code	Description	Chemical Formula	CH:DCPD
DCPD	Dicalcium Phosphate Dihydrate	CaHPO_4_·2H_2_O	0:100
CH	Chitosan scaffold	(C_6_H_11_NO_4_)_n_	100:0
20-DCPD	Mineral loaded scaffold	-	80:20
30-DCPD	Mineral loaded scaffold	-	70:30
40-DCPD	Mineral loaded scaffold	-	60:40
50-DCPD	Mineral loaded scaffold	-	50:50

**Table 2:** Rietveld analysis, Bragg’s law and Scherrer’s equation were utilised to determine diffraction plane indexes, crystallite size and crystallinity of the unloaded and DCPD mineral-loaded freeze-dried chitosan scaffolds.

Sample	Diffraction Plane (h k l)		Crystallite Size (nm)	Crystallinity (%)
	(0 2 0)	(0 4 0)		
CH	-	-	0.9059	0.1104
20-DCPD	11.53°	20.64°	2.2404	20.6399
30-DCPD	11.93°	21.04°	11.2882	29.3773
40-DCPD	11.53°	20.64°	25.9477	38.7656
50-DCPD	11.14°	20.44°	26.4008	69.8635
DCPD	10.97°	22.75°	41.2679	78.5123

**Table 3:** Mechanical property data was obtained from tensile testing freeze-dried scaffolds (n = 3).

	CH	20-DCPD	30-DCPD	40-DCPD	50-DCPD
**Young’s Modulus (kN/m^2^)**	5.38 ± 0.03	7.26 ± 0.06	20.92 ± 0.11	22.73 ± 2.10	25.50 ± 0.54
**Tensile Strength (kPa)**	7.07 ± 0.03	9.86 ± 0.05	26.84 ± 0.08	23.21 ± 0.63	27.13 ± 0.25

**Table 4:** Zeta potential measurements of the unloaded and Dicalcium Phosphate Dihydrate (DCPD) loaded freeze-dried chitosan scaffold suspensions.

Sample	Zeta Potential (mV)	Standard Deviation
DCPD	−12.44	0.41
CH	+43.47	0.35
20-DCPD	+39.57	0.31
30-DCPD	+34.53	0.38
40-DCPD	+28.23	0.25
50-DCPD	+20.23	0.47

## Data Availability

The datasets used and/or analysed during the current study are available from the corresponding author on reasonable request.
